# Screening of Drug Metabolizing Enzymes for the Ginsenoside Compound K *In Vitro*: An Efficient Anti-Cancer Substance Originating from *Panax Ginseng*

**DOI:** 10.1371/journal.pone.0147183

**Published:** 2016-02-04

**Authors:** Jian Xiao, Dan Chen, Xiu-Xian Lin, Shi-Fang Peng, Mei-Fang Xiao, Wei-Hua Huang, Yi-Cheng Wang, Jing-Bo Peng, Wei Zhang, Dong-Sheng Ouyang, Yao Chen

**Affiliations:** 1 Department of Clinical Pharmacology, Xiangya Hospital, Central South University, Changsha, Hunan, China; 2 Institute of Clinical Pharmacology, Central South University, Changsha, Hunan, China; 3 Department of Hepatology and Infectious Diseases, Xiangya Hospital, Central South University, Changsha, Hunan, China; 4 Health Management Center, Xiangya Hospital, Central South University, Changsha, Hunan, China; 5 Department of Pharmacy, Xiangya Hospital, Central South University, Changsha, Hunan, China; Hunter College of The City University of New York, UNITED STATES

## Abstract

Ginsenoside compound K (CK), a rare ginsenoside originating from *Panax Ginseng*, has been found to possess unique pharmacological activities specifically as anti-cancers. However, the role of cytochrome P450s (CYPs) in the metabolism of CK is unclear. In this study, we screened the CYPs for the metabolism of CK *in vitro* using human liver microsomes (HLMs) or human recombinant CYPs. The results showed that CK inhibited the enzyme activities of CYP2C9 and CYP3A4 in the HLMs. The *K*_*m*_ and *V*_*max*_ values of CK were 84.20±21.92 μM and 0.28±0.04 nmol/mg protein/min, respectively, for the HLMs; 34.63±10.48 μM and 0.45±0.05 nmol/nmol P450/min, respectively, for CYP2C9; and 27.03±5.04 μM and 0.68±0.04 nmol/nmol P450/min, respectively, for CYP3A4. The *IC*_*50*_ values were 16.00 μM and 9.83 μM, and *K*_*i*_ values were 14.92 μM and 11.42μM for CYP2C9 and CYP3A4, respectively. Other human CYP isoforms, including CYP1A2, CYP2A6, CYP2D6, CYP2E1, and CYP2C19, showed minimal or no effect on CK metabolism. The results suggested that CK was a substrate and also inhibitors for both CYP2C9 and CYP3A4. Patients using CK in combination with therapeutic drugs that are substrates of CYP2C9 and CYP3A4 for different reasons should be careful, although the inhibiting potency of CK is much poorer than that of enzyme-specific inhibitors.

## Introduction

*Panax Ginseng* is a complementary and alternative medicine (CAM) that has many pharmacological activities, such as maintaining physical vitality and boosting resistance to stress and aging [1]. *Panax Ginseng* is widely used in clinics for cancer prevention, erectile dysfunction, and enhanced physical functions, among others [2]. The main active constituents of *Panax Ginseng* are ginsenosides, and to date, more than 150 naturally derived ginsenosides have been obtained from *Panax Ginseng* [3]. The main constituents comprising more than 80% of the content of *Panax Ginseng* are Rb1, Rb2, Rc, Rg1, and Re. The rest are known as rare ginsenosides, which account for only a small portion of the total saponins, and they have unique pharmacological activities [4].

**Fig 1:**
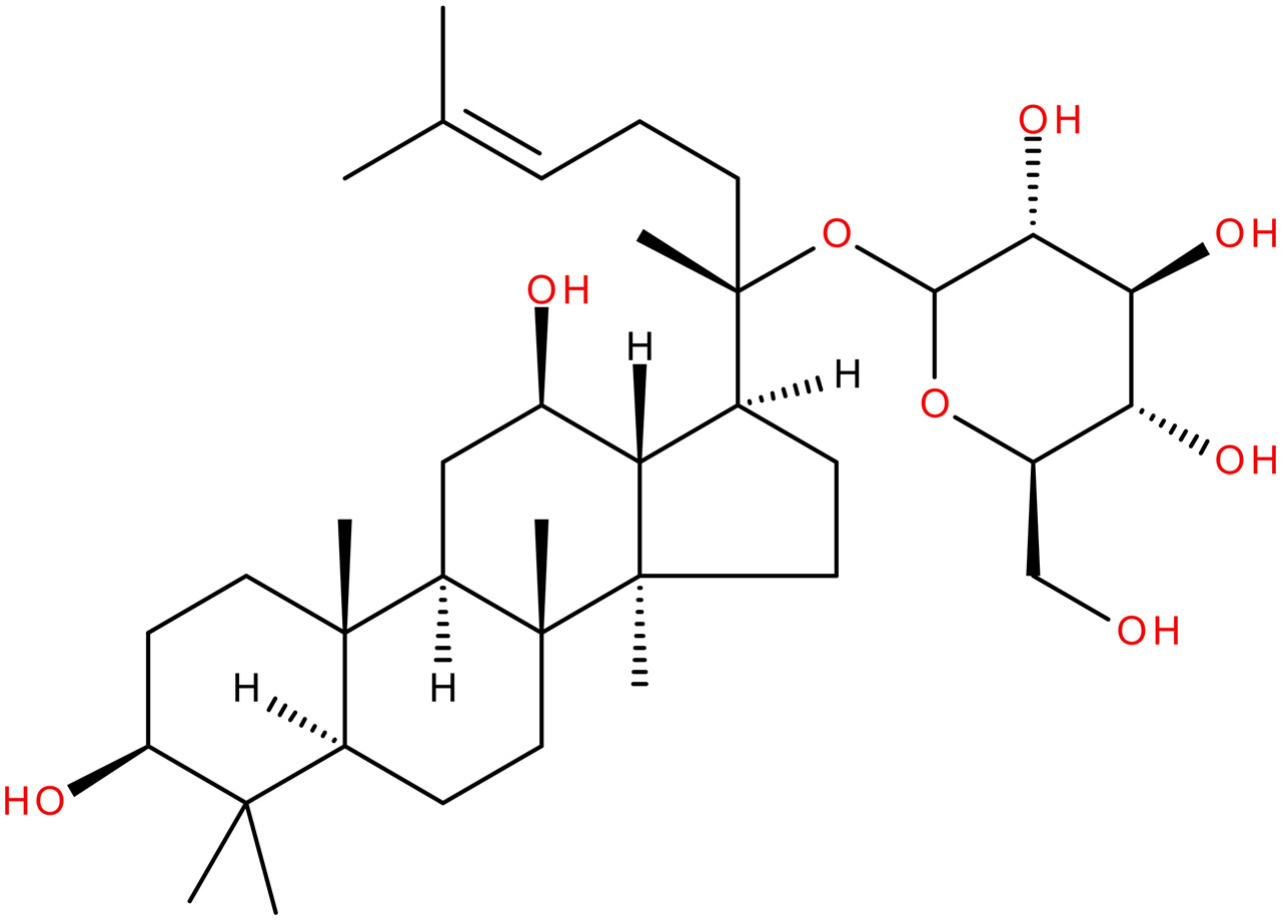
The molecular structure of CK.

Ginsenoside compound K (CK), or 20-O-β-(D-glucopyranosyl)-20(S)-protopanaxadiol (The molecular structure is shown in Fig 1), is naturally absent. It belongs to the rare ginsenoside, which is transformed from ginsenosideRb1 and Rb2 by intestinal bacteria [5]. Ginsenosides are poorly absorbed from the gut while their metabolite CK is absorbed. As the final form, CK playing a pharmacological role has caused growing interest. CK can resist all kinds of tumor cells and promote cell apoptosis, such as colorectal cancer cells, gastric cancer cells, lung cancer cells, and multiple myeloma [1, 6, 7]. CK increases the resistance to immunity function to extend the transplant survival time in heart transplantation, has an anti-inflammatory function, resists skin aging, and protects the myocardium [1, 8].

The unique biological activity of CK has attracted much attention, and thus it has a wide application prospect. The current use of CAM by patients is increasing [9]. The concomitant use of CAM and therapeutic drugs could lead to serious safety issues in patients. CAM has the potential to cause pharmacokinetic interactions with therapeutic drugs. It can either increase or decrease the plasma levels of therapeutic drugs and result in unexpected toxicities or reduced efficacy [10].

Most pharmacokinetic CAM–drug interactions involve drug metabolizing cytochrome P450 (CYP) enzymes [11, 12]. The CYPs are a large family of drug-metabolizing enzymes that play a critical role in phase I drug metabolism. Moreover, most of the endogenous and exogenous substances are the substrates of CYPs. The major CYPs involved in the metabolism of most drugs are CYP1A2, CYP2A6, CYP2C9, CYP2D6, CYP2E1, CYP2C19, and CYP3A4, which account for more than 90% of the metabolism of endogenous and exogenous substances [13].

CK is one of the most frequently used CAM for many diseases in clinics. As co-administration cannot be avoided, how to prevent CK–drug interactions has become a main issue among patients. To provide rational advice for the usage of drugs, we examined the CYP enzymes responsible for the metabolism of CK in the present study.

## Methods

### Enzymes and chemicals

The enzymes and chemicals ware prepared according to our previous study with some modifications [14]. Pooled human liver microsomes, recombinant human CYP enzymes, and NADPH were purchased from Cypex Ltd. (U.K.) and stored at -80°C until use. Ginsenoside compound K (CK, C_36_H_62_O_8_, MW:622.873, assay≥99.2%, Lot: P1201-1) was provided by Zhejiang Hisun Pharmaceutical Company Limited (Zhejiang, China). Furafylline, tranylcypromine, ketoconazole,sulfaphenazole, quinidine, chlormethiazole hydrochloride, and ticlopidine hydrochloride were purchased from the National Institutes for Food and Drug Control (Beijing, China). Phenacetin, coumarin, midazolam, tolbutamide, S-Mephenytoin, metoprolol, chlorzoxazone, and the standards for their metabolites, including acetaminophen, 7-hydroxyl coumarin, 1-hydroxyl midazolam, 4-hydroxyl tolbutamide, 4-hydroxyl mephenytoin, α-hydroxyl metoprolol, 6-hydroxyl chlorzoxazone, and irbesartan (the internal standard), were purchased from Sigma-Aldrich (Shanghai, China). All other chemicals and solvents were of high-performance liquid chromatography (HPLC) grade.

### Apparatus and operation conditions

The concentrations of the CYP substrates and their metabolites were quantified according to the method reported in our previous study with some modifications [14]. A Waters 2695 separation module HPLC system coupled to a Quattro micro^™^ API triple quadrupole tandem mass spectrometer (Waters Corp., Milford, Massachusetts, USA) with an electrospray ionization source was used. The samples were separated on a HyPURITY C_18_ column (150 mm × 2.1 mm, 5 μm, Thermo, USA). The mobile phases consisted of 20 mM ammonium formate and acetonitrile at a ratio of 60:40. Aliquots of 20 μL were injected at a mobile phase flow rate of 0.3 mL/min. Multiple reaction monitoring was conducted in the positive mode, except for CK that was conducted in the negative mode (parent ion m/z 621.4 to daughter ion m/z 160.8). The other transitions were the same as those reported in our previous study [14]. The mass spectra of the metabolites formed in the incubations were identical to those of the corresponding authentic standards.

### General incubation conditions

The method was conducted according to our previous study with some modifications [14]. The CYP isoform-specific probe reactions used were phenacetin O-deethylation (for CYP1A2), coumarin 7-hydroxylation (for CYP2A6), tolbutamide 4-hydroxylation (for CYP2C9), metoprolol α-hydroxylation (for CYP2D6), chlorzoxazone 6-hydroxylation (for CYP2E1),S-Mephenytoin 4-hydroxylation (for CYP2C19), and midazolam 1-hydroxylation (for CYP3A4). The kinetic study of CK was conducted with HLMs. A 0.2 mL incubation mixture that consisted of CK (as a substrate), the HLMs (0.5 mg/mL) or CYP isoforms (10 pmol), and 0.1 M sodium phosphate buffer (pH 7.4) was pre-warmed for 5 min at 37°C. The reaction was initiated by the addition of 1 mg/mL triphosphopyridine nucleotide (NADPH). All the reagents were dissolved in methanol, and the final solvent concentration in all incubations (including the controls) was 1%. The final incubations were performed in a shaking water bath (37°C) for 30 min. The reactions were stopped by adding 0.2 mL ice-cold acetonitrile, which contains irbesartan (114.9 ng/mL) as the internal standard. The samples were vortexed for 5 min. After centrifugation (12000×g for 10 min), the supernatants were transferred, and aliquots of 20 μL were injected into the HPLC-MS/MS system for analysis.

### Kinetic analysis of CK

The kinetic parameters for the metabolism of CK were determined by incubating increasing concentrations of CK (10–100 μM) at 37°C with the HLMs and NADPH under the incubation conditions as described in detail previously [14]. The equation of CK reaction velocity (*V*) in the HLMs or CYP isoforms is expressed as *V = (C*_*0*_*–C*_*t*_*)/*T*/C*_*p*_, where *C*_*0*_and *C*_*t*_ are the initial and final concentrations of CK in the incubation solution, respectively, T is the incubation time (min), and *Cp* is the protein concentration (mg/mL or nmol). All values were expressed as the mean±standard deviation (SD). The mean intrinsic clearance rate (*CL*_*int*_) for the *in vitro* incubation was estimated using *V*_*max*_*/K*_*m*_.

### Specific CYP isoforms screened for the CK metabolism

To screen the specific CYP isoform responsible for the CK metabolism, we determined the inhibitory effect of specific inhibitors on the metabolism of CK in the HLMs as described in detail previously [14]. Inhibitors including furafylline (for CYP1A2), tranylcypromine (for CYP2A6), sulfaphenazole (for CYP2C9), quinidine (for CYP2D6), chlormethiazole (for CYP2E1), ticlopidine (for CYP2C19), and ketoconazole (for CYP3A4) were separately incubated with CK (50 μM), the HLMs, and NADPH under the same incubation conditions as mentioned above.The concentrations of the inhibitors used were approximately at their respective *IC*_*50*_ values according to previous reports [15–18]. The inhibitory effects of the above specific inhibitors on the metabolic clearance rate of CK were evaluated separately to screen the CYP isoforms responsible for the CK metabolism. The relative activity of the CYP isoforms was calculated by dividing the peak area of CK when incubated with the inhibitor by that of CK from the negative controls.

### Inhibition studies on *IC*_*50*_ determination

CK (10–100 μM) and a single CYP isoform-specific substrate (with a concentration similar to its corresponding *K*_*m*_value) were used to determine the inhibitory effect of CK on specific CYP isoforms as described in detail previously [14]. Substrates including phenacetin, coumarin, tolbutamide, metoprolol, chlorzoxazone, S-Mephenytoin, and midazolam were used at concentrations of 10, 5, 100, 7.5, 40, 100, and 5 μM, respectively. All incubation conditions were the same as mentioned above. The inhibitory effects on the CYP isoforms were investigated individually by incubating the HLMs in the absence or presence of CK. The incubation solution with the solvent that was used to dissolve CK was regarded as the negative control, and the solutions containing the specific inhibitors mentioned above were considered the positive controls.

### Determination of *K*_*i*_

In the *IC*_*50*_ determination experiments, CK markedly inhibited CYP2C9 and CYP3A4, whereas its effect on CYP1A2, CYP2A6, CYP2D6, CYP2E1 and CYP2C19 was minimal. Therefore, Dixon plots for the inhibition of CYP2C9 and CYP3A4 were determined by incubating the substrate probe at multiple concentrations with or without the test inhibitor with the HLMs and cofactors. The inhibition data obtained from the pilot experiments were used as a guide to generate the appropriate probe substrate and test inhibitor concentrations for the determination of the *K*_*i*_ values for each CYP isoform. The isoform-specific probe substrate concentrations used were 25–200 μM tolbutamide for CYP2C9 and 5–50 μM midazolam for CYP3A4. The CK concentrations used were 10–100 μM.

### Determination of *K*_*i*_^*’*^

According to Cornish-Bowden *et al*.[19], a Dixon plot is limited by the fact that it does not distinguish unambiguously between competitive and mixed inhibitors and, for mixed or uncompetitive inhibitors, it provides no measure of *K*_*i’*_, the dissociation constant of the EIS complex. Therefore, we used S/V versus [I] plots to determine the *K*_*i’*_ values for the inhibition of CYP2C9 and CYP3A4. When *K*_*i*_>*K*_*i*_’, the intersection was above the [I] axis in the plot of S/V against [I] and below it in the Dixon plot; the opposite was true if *K*_*i*_<*K*_*i*_*’*. In the special case where *K*_*i*_ = *Ki’* (simple non-competitive inhibition), the intersections occurred on the [I] axis in both plots. The asymptotic cases of competitive and uncompetitive inhibition can be generated by inserting the conditions *K*_*i’*_ to ∞ for competitive inhibition, or *K*_*i*_ to ∞ for uncompetitive inhibition [19].

### Calculation of the enzyme kinetics and statistical method

As described in detail previously [14], to determine the major enzymes responsible for the CK metabolism in HLMs, the metabolic clearance rate of the incubation solution without any specific inhibitor for CK was considered as 100%. The effects of the specific inhibitors on the metabolic clearance rate of CK were evaluated with SPSS one-way analysis of variance (SPSS Inc., Chicago, IL, USA). P<0.05 was considered significant in all cases. The apparent kinetic parameters of CK (*K*_*m*_ and *V*_*max*_) were determined by fitting the Michaelis–Menten equation using the GraphPad Prism Enzyme Kinetic 5 Demo software (GraphPad Co. Ltd, San Diego, CA, USA). The equation is expressed as *V = V*_*max*_*[S]/(K*_*m*_*+[S])*, where *K*_*m*_ is the substrate concentration at which the reaction velocity is 50% of *V*_*max*_. To determine the inhibition of the CYP isoforms, the activity of each CYP isoform was calculated using the metabolic clearance rate of its corresponding probe substrate. The metabolic clearance rate of the probe substrate was considered 100% when no specific inhibitor and CK were added to the incubation assay. The *IC*_*50*_*s* were determined by analyzing the plot of the logarithm of the inhibitor concentration versus the percentage of activity remaining after inhibition using the SPSS software for Windows (version 11.5, SPSS, Chicago, IL). To calculate the *K*_*i*_ values, the inhibition data were fit to different models of enzyme inhibition (competitive, noncompetitive) by nonlinear least-squares regression analysis using the GraphPad Prism 5 software (GraphPad Co. Ltd), *K*_*i*_ values were calculated with the use of nonlinear regression according to the equation *v* = (*V*_*max*_*S*)/(*K*_*m*_(1+*I/K*_*i*_)+*S*) for competitive inhibition and equation *v* = (*V*_*max*_*S*)/(*K*_*m*_+*S*)(1+*I/K*_*i*_) for noncompetitive inhibition, where *I* is compound concentration, *K*_*i*_ is the inhibition constant, *S* is the substrate concentration, and *K*_*m*_ is the substrate concentration at half of the maximum velocity (*V*_max_) of the reaction [20]. *K*_*i*_*’ values* were calculated using the secondary plot of slopes of the S/V versus [I] [19].

## Results

### Kinetic analysis of CK

**Fig 2:**
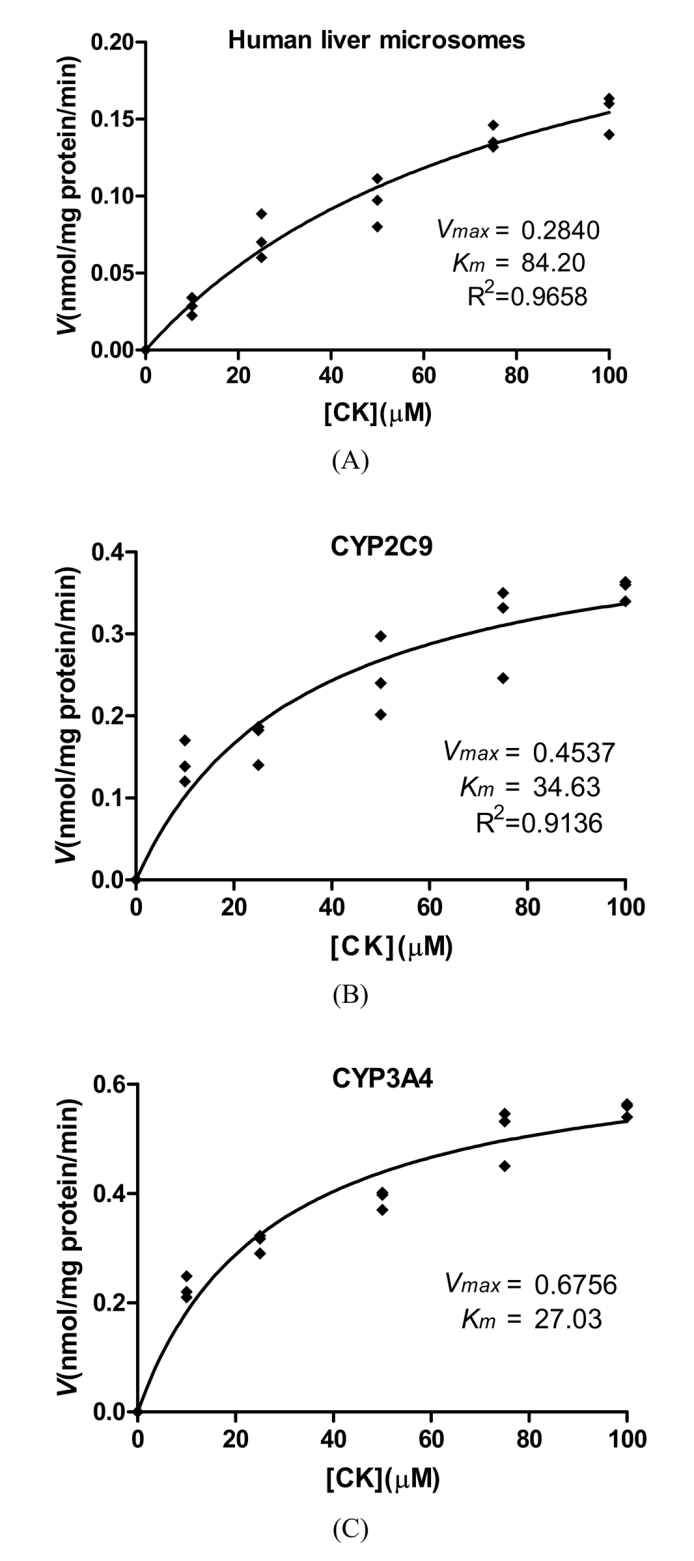
Concentration-velocity curve of CK metabolism after incubation with HLMs (A), recombinant CYP2C9 (B) and recombinant CYP3A4 (C). Note: The incubation conditions are described in the materials and methods section. The curve was automatically fitted using nonlinear regression and the Michaelis–Menten equation. Data were obtained in triplicate.

Fig 2A, 2B and 2C show the metabolism of CK after incubation with the HLMs, CYP2C9, and CYP3A4. The kinetic plots indicated that the *K*_*m*_ and *V*_*max*_ values were 84.20±21.92 μM and 0.28±0.04 nmol/mg protein/min for the HLMs, 34.63±10.48 μM and 0.45±0.05 nmol/nmol P450/min for CYP2C9, 27.03±5.04 μM and 0.68±0.04 nmol/nmol P450/min for CYP3A4, respectively. The *in vitroCL*_*int*_ values of CK in the HLMs, CYP2C9, and CYP3A4 were 0.0033 mL/mg protein^-1^·min^-1^, 0.0130 mL/nmol P450^-1^·min^-1^, and 0.0252 mL/nmol P450^-1^·min^-1^, respectively.

### Specific CYP isoforms for the metabolism of CK

**Fig 3:**
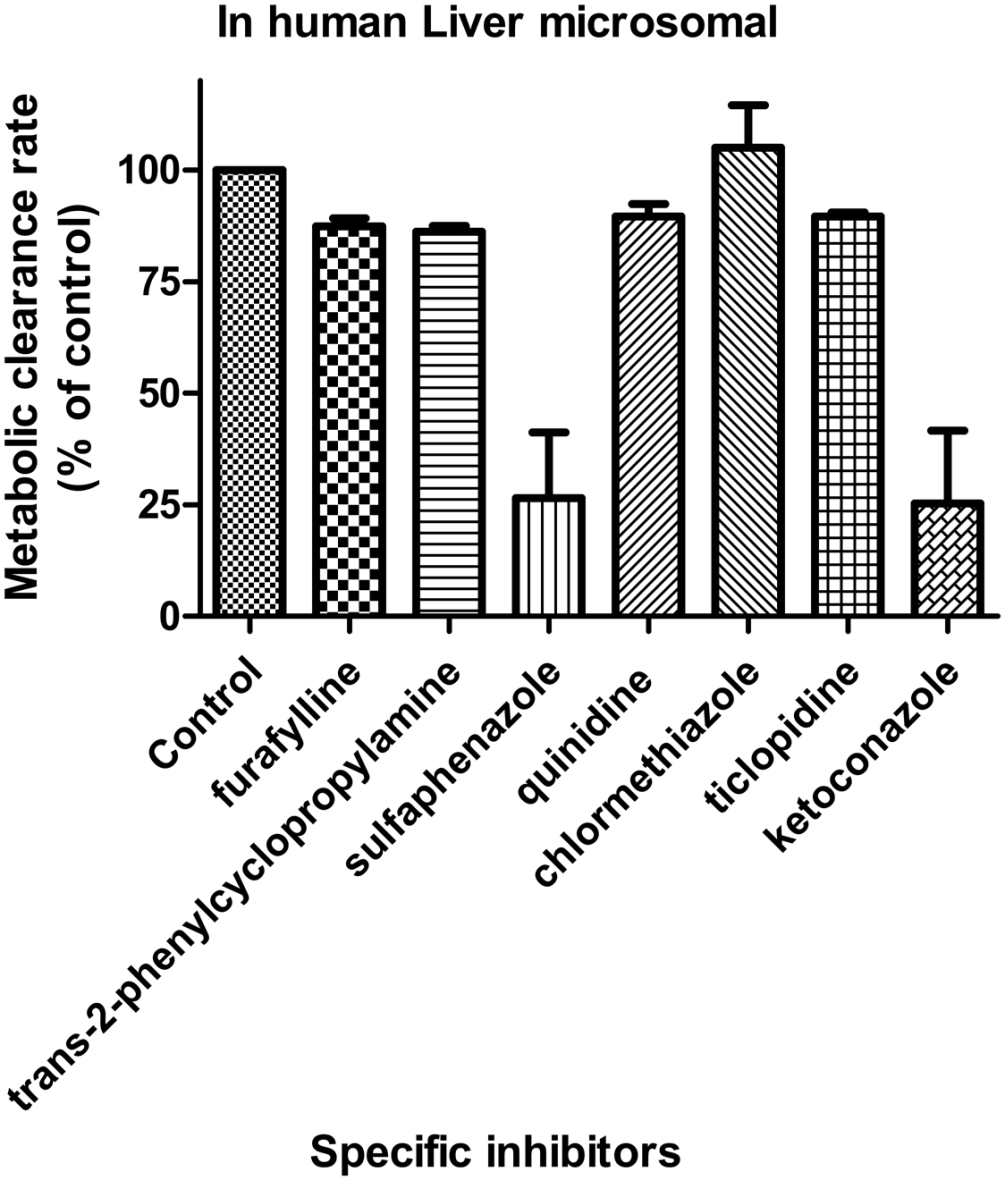
Effect of specific inhibitors on CYP-mediated CK (50 μM) metabolism in HLMs. Note: Furafylline, tranylcypromine, sulfaphenazole, quinidine, chlormethiazole, ticlopidine, and ketoconazole were used as the specific inhibitors for CYP1A2, CYP2A6, CYP2C9, CYP2D6, CYP2E1, and CYP2C19, respectively. The incubation conditions are described in the materials and methods section. Each data point represents the average of triple determinations and error bars. In the presence of sulfaphenazole (1 μM) and ketoconazole (1 μM), the metabolic clearance rate of CK decreased to 73.5% and 74.6% of that of the control, respectively, whereas the other inhibitors had no significant inhibitory effects on the metabolism of CK.

**Fig 4:**
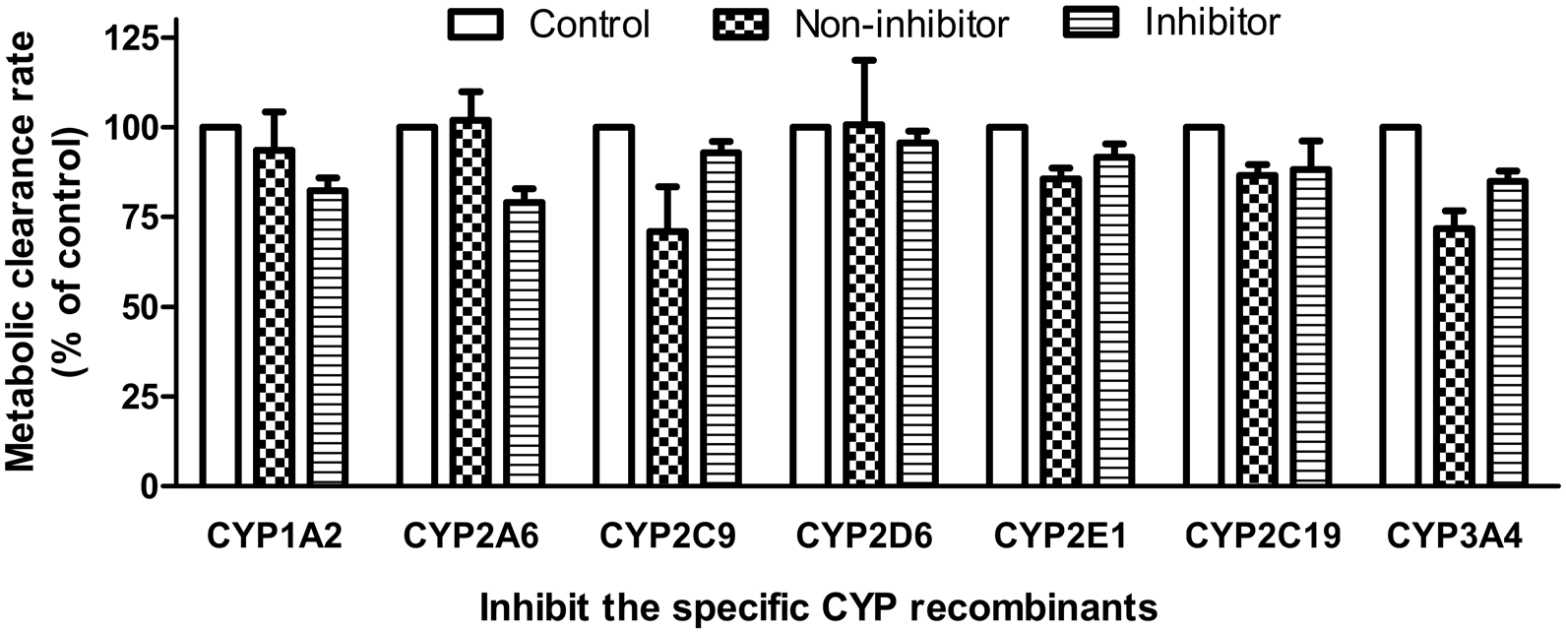
Effect on the metabolic clearance rate (MCR) of CK under the inhibition of recombinant CYP2C9 and CYP3A4. Note: About 50 μM of CK was incubated with the CYP recombinants and cofactors in the absence (control) or presence of the inhibitors by sulfaphenazole (1 μM) and ketoconazole (1 μM), respectively. Each point represents the average of triplicate incubations. The MCRs of CK were significantly decreased compared with those of the control for CYP3A4 and CYP2C9.

The inhibitory effects of the CYP specific inhibitors on the metabolic clearance rate of CK in the HLMs are shown in Fig 3. The concentrations of furafylline, tranylcypromine, sulfaphenazole, quinidine, ticlopidine, and ketoconazole were 1 μM, except for chlormethiazole at 5 μM. The concentrations were selected on the basis of previously reported *IC*_*50*_ or *K*_*i*_ values for the CYP isoforms to ensure adequate inhibitory selectivity and maximal inhibitory potency. In the presence of sulfaphenazole and ketoconazole, the metabolic clearance rate (MCR) of CK decreased to 73.5±20.9% and 74.6±28.1% of that of the control, respectively (Fig 3). However, other inhibitors had no obvious inhibitory effects on the metabolism of CK. The screened enzymes were further confirmed by human recombinant CYPs using the specific inhibitors. The MCRs of CK decreased to 22.0% of that of the control for CYP2C9 and to 13.2% of that of the control for CYP3A4 (Fig 4). The results indicated that CYP2C9 and CYP3A4 were the major enzymes responsible for the metabolism of CK *in vitro*.

### Estimation of *IC*_*50*_*s*

**Fig 5:**
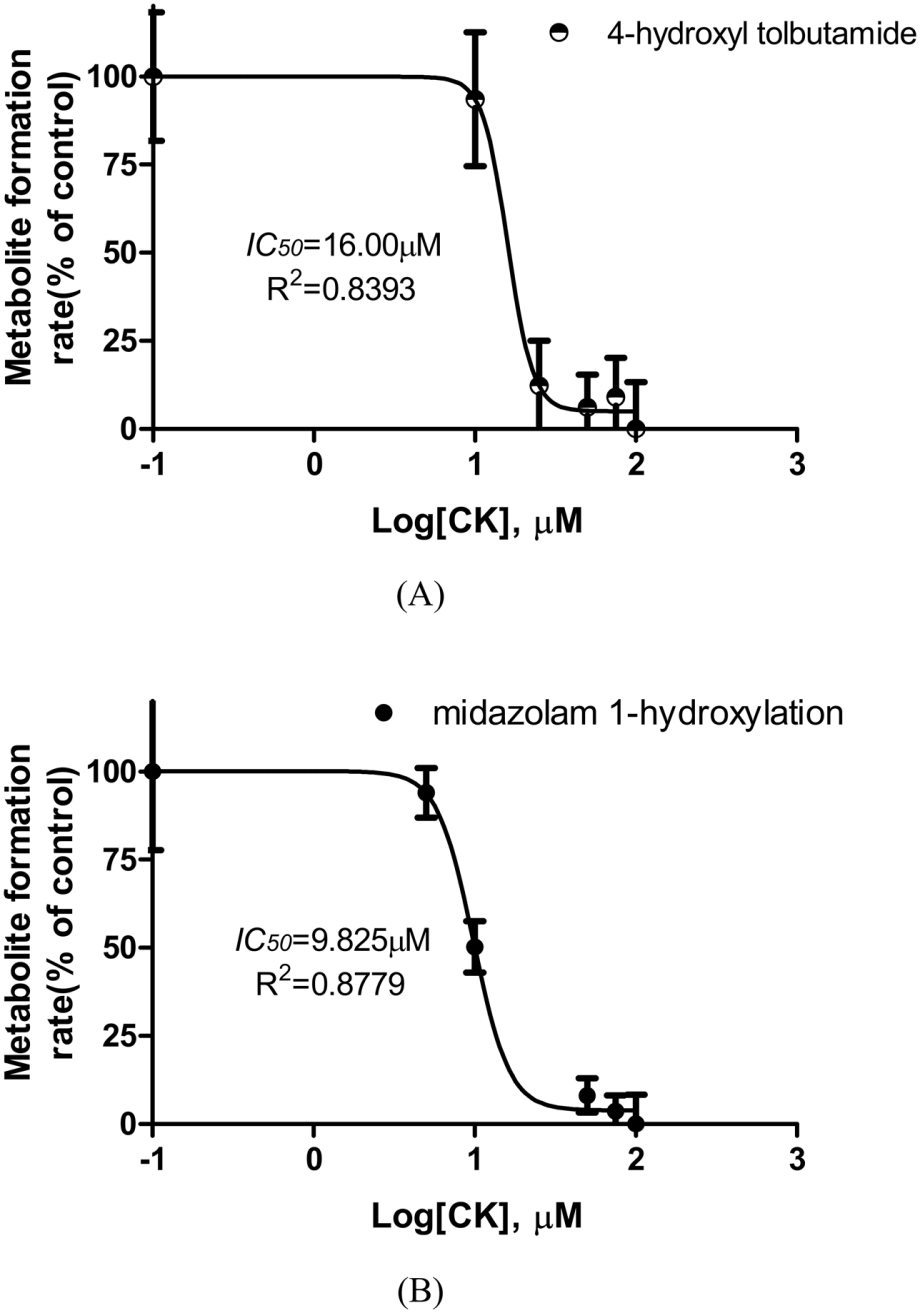
Effect of CK on the metabolic reactions of the CYP2C9 (A) and CYP3A4 (B) specific substrates in HLMs. Note: The incubation conditions are described in the materials and methods section. Each data point represents the average of triple determinations and error bars.

The inhibitory effects of multiple concentrations of CK (10–100 μM) on the activity of each CYP isoform determined by the metabolism of a single concentration of isoform-specific probe were tested with the HLMs (or expressed CYPs when needed). CK showed the potent inhibition of CYP2C9 (tolbutamide 4-hydroxylation) and CYP3A4 (midazolam 1-hydroxylation)(Fig 5), with *IC*_*50s*_ of 16.00 μM and 9.83 μM, respectively. The inhibitory effect of CK on the activity of CYP1A2, CYP2A6, CYP2D6, CYP2E1 and CYP2C19 was negligible.

### Estimation of *K*_*i*_ values

**Fig 6:**
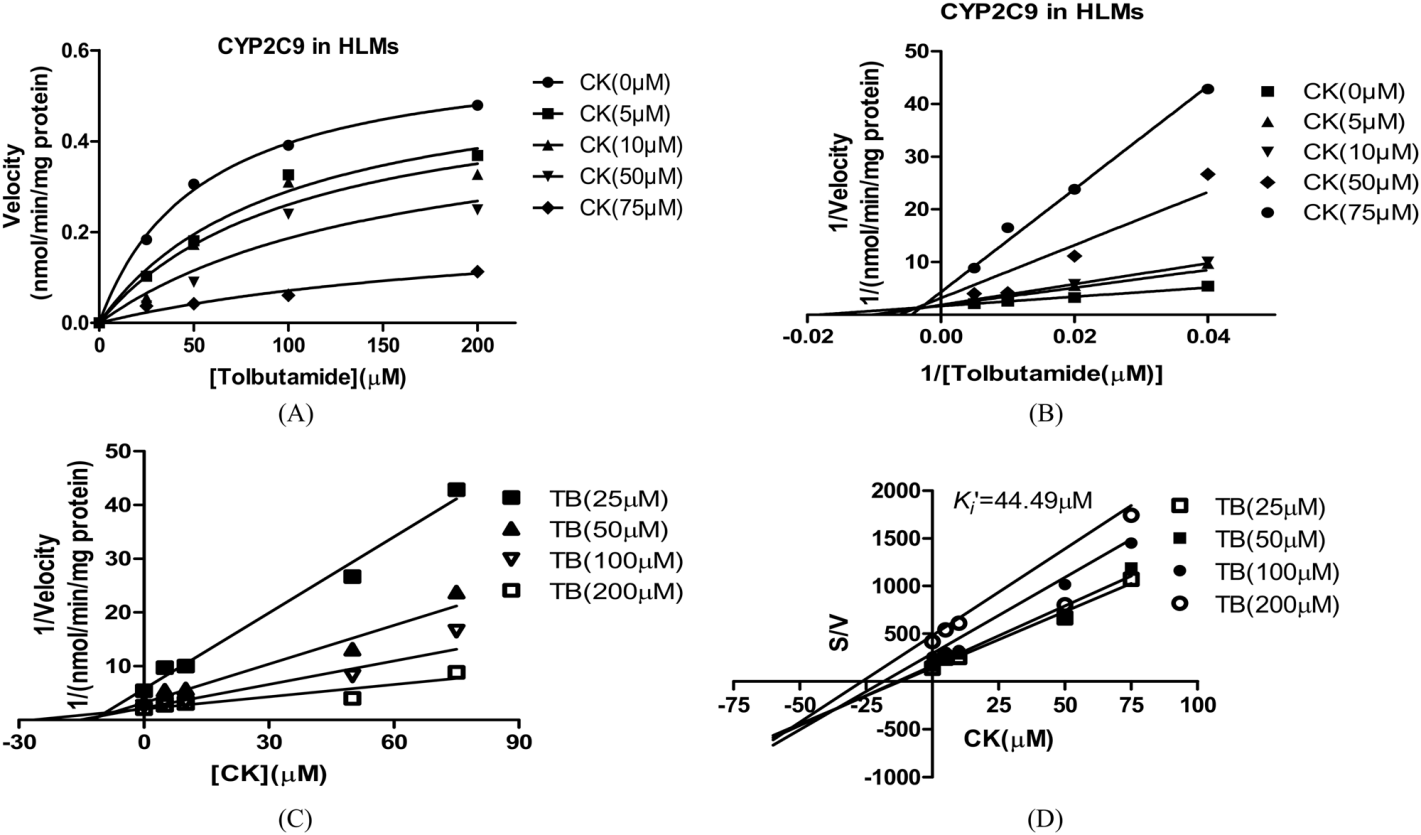
A nonlinear regression analysis curves of velocity (nmol/min/mg protein) enzyme activity for tolbutamide (μM) assuming Michaelis-Menten kinetics (A), representative Lineweaver-Burk plots (B) and Dixon plots (C) of the effect of CK on tolbutamide 4-hydroxylation formation and S/V versus [I] plots (D)in human liver microsomes, Note: The inhibition of CYP2C9 activity is best described as a mixed type inhibition mechanism by different concentrations of CK (10–100 μM) in HLM incubations (0.5 mg·mL^-1^ protein). The data points are the mean values of triplicate incubations. TB, tolbutamide.

**Fig 7:**
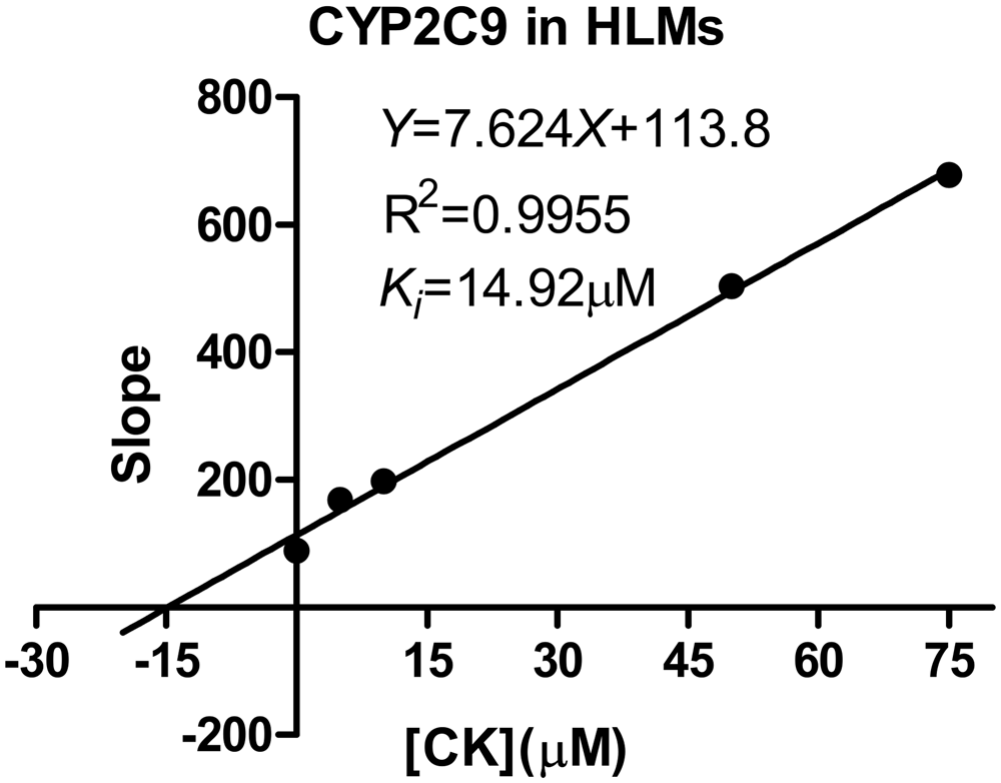
Secondary plot of CYP2C9 activity using the slopes of the primary Lineweaver–Burk plots versus concentrations of CK. Note: Nonlinear regression analysis of the tolbutamide 4-hydroxylation versus the substrate concentration was performed to obtain the *K*_*i*_ values. Each point represents the mean of triplicate determinations.

**Fig 8:**
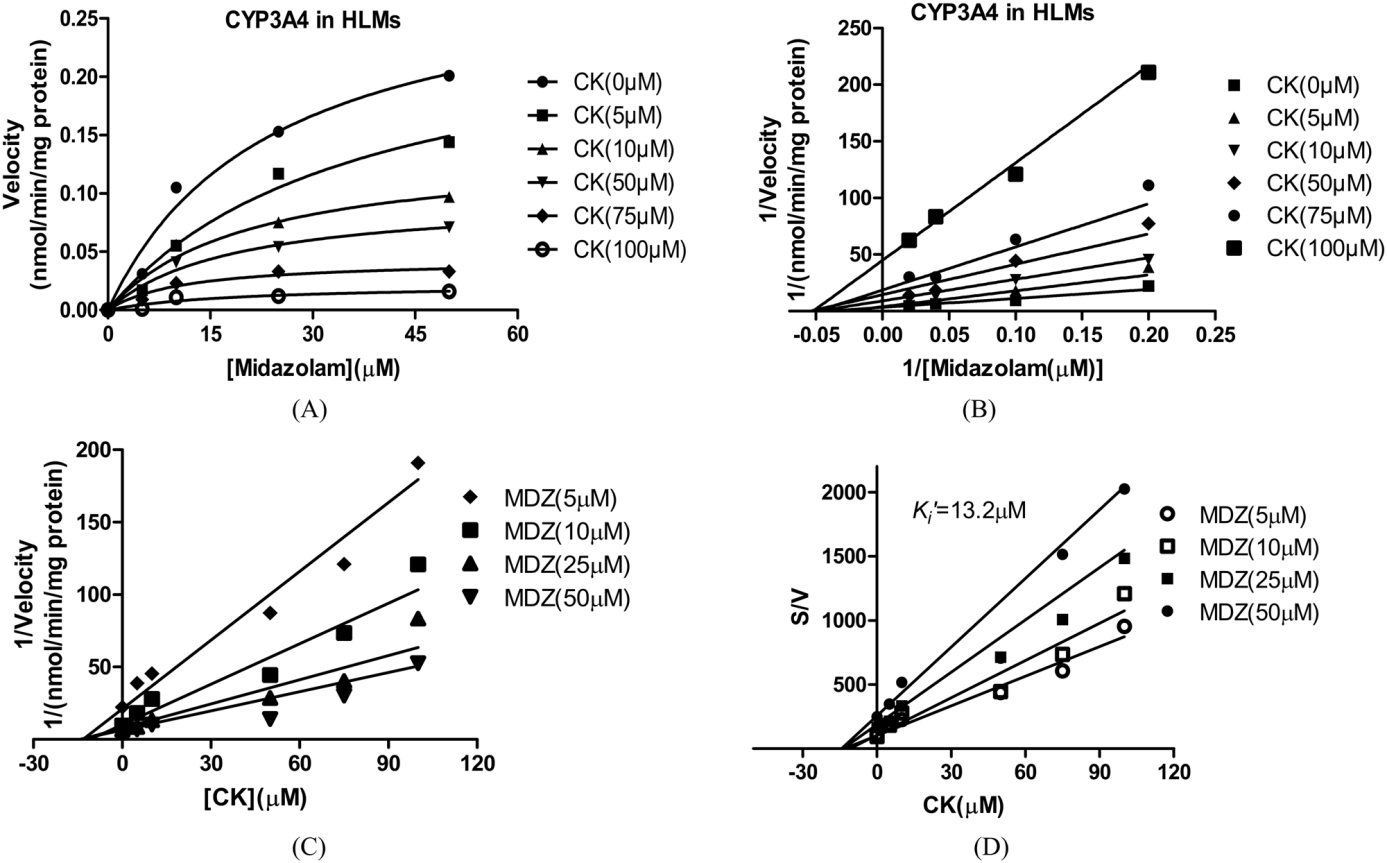
A nonlinear regression analysis curves of velocity (nmol/min/mg protein) enzyme activity for midazolam (μM) assuming Michaelis-Menten kinetics (A), representative Lineweaver-Burk plots (B) and Dixon plots (C) of the effect of CK on midazolam 1-hydroxylation formation and S/V versus [I] plots (D) in human liver microsomes. Note: The inhibition of CYP3A4 activity is best described as a noncompetitive inhibition by different concentrations of CK (10–100 μM) in HLM incubations (0.5 mg·mL^-1^ protein). The data points are the mean values of triplicate incubations. MDZ, midazolam.

**Fig 9:**
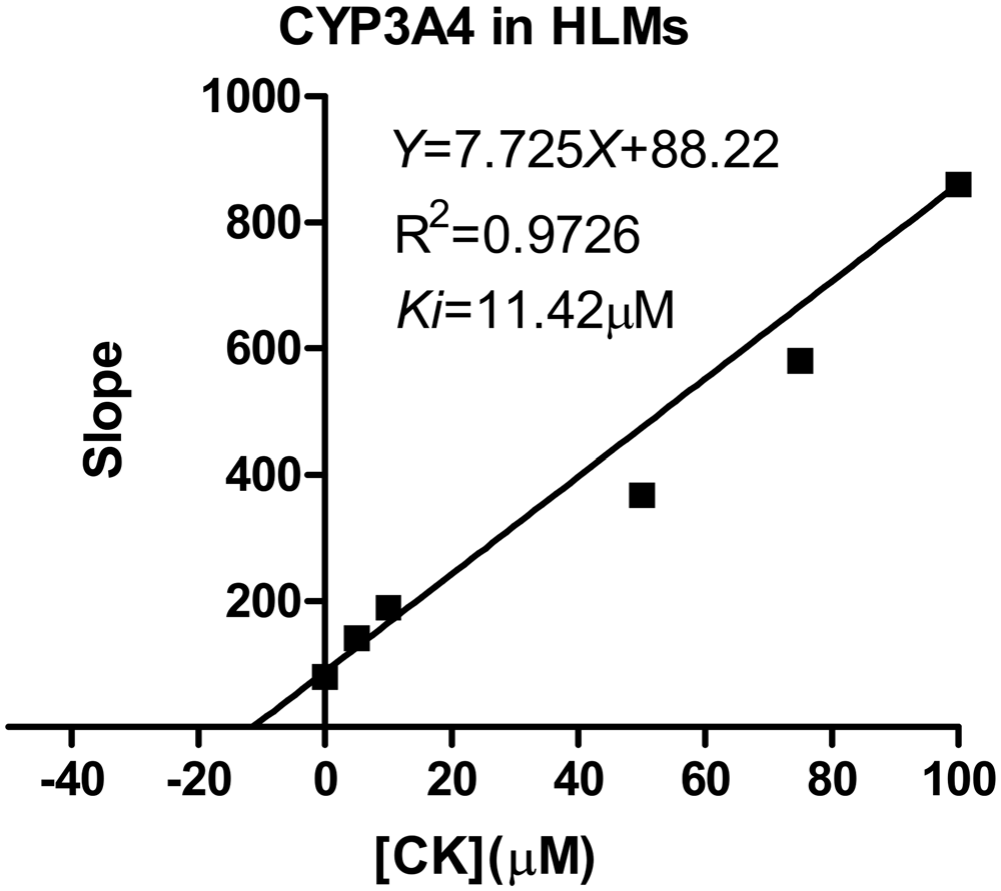
Secondary plot of CYP3A4 activity using the slopes of the primary Lineweaver–Burk plots versus concentrations of CK. Note: Nonlinear regression analysis of the CK versus the substrate concentration was performed to obtain the *K*_*i*_ values. Each point represents the mean of triplicate determinations.

For CYP2C9, the *K*_*i*_ values were determined using tolbutamide as the probe substrate. The *V*_*max*_ and *K*_*m*_ values estimated using a nonlinear regression model for the competitive enzyme inhibition of CYP2C9-catalyzed tolbutamide 4-hydroxylation in the HLMs, the representative Lineweaver-Burk plots (Fig 6) and the secondary plot of CYP2C9 activity using the slopes of the primary Lineweaver–Burk plots versus concentrations of CK show that *K*_*i*_of CK were 14.92 μM (Fig 7) [20]. The representative Dixon plots of the effect of CK on tolbutamide 4-hydroxylation formation and S/V versus [I] plots (Fig 6) suggested that the inhibition data fit well to a mixed type of inhibition [19]. For CYP3A4, the *K*_*i*_ values were determined using midazolam as the probe substrate. The *V*_*max*_ and *K*_*m*_ values estimated using a nonlinear regression model for the competitive enzyme inhibition of CYP3A4-catalyzed midazolam 1-hydroxylation in the HLMs (Fig 8). The representative Lineweaver-Burk plots (Fig 8) and the secondary plot of CYP2C9 activity using the slopes of the primary Lineweaver–Burk plots versus concentrations of CK show that *K*_*i*_of CKvalues were 11.42 μM (Fig 9) [20]. The representative Dixon plots of the effect of CK on midazolam 1-hydroxylation formation and S/V versus [I] plots (Fig 8) showed that the inhibition data fit well to a noncompetitive inhibition [19].

## Discussion

Currently, the concomitant use of CAM and therapeutic drugs are popular. CAM has the potential to cause pharmacokinetic and dynamic interactions with therapeutic drugs. Therefore, the decreased or increased plasma levels of these drugs could result in a lower therapeutic efficacy or a higher risk of toxicity [9, 10]. Especially for anti-cancer drugs, which have narrow therapeutic windows, pharmacokinetic interactions could easily lead to clinically relevant effects [9]. CK is a CAM that has a prospective application for treating cancer [5–7]. Therefore, screening the drug-metabolizing enzymes of CK may provide useful information for avoiding CK–drugs interactions.

The CYP superfamily comprises important phase Ι drug-metabolizing enzymes that oxidize more than 90% of the current therapeutics drugs. Over 90% of human drug oxidation can be attributed to CYP1A2, CYP2A6, CYP2C9, CYP 2C19, CYP2E1, CYP2D6, and CYP3A4 [12]. Drugs competing with the same metabolizing enzyme can lead to drug–drug interactions, resulting in altered efficacy or toxicity in individuals [13].

In the present study, our data suggested that CYP2C9 and CYP3A4 were the major enzymes involved in the metabolism of CK *in vitro*, not the substrates of CYP1A2, CYP2A6, CYP2D6, CYP2E1 and CYP2C19. Furthermore, our study showed that CYP2C9 and CYP3A4 were sensitive to the inhibition by CK, and their *IC*_*50*_ valueswere 16.00 μM and 9.83 μM, respectively. CYP1A2, CYP2A6, CYP2D6, CYP2E1, and CYP2C19 were hardly sensitive to the inhibition of CK (*IC*_*50s*_ were all greater than 100 μM). These findings are partly supported by the study of Hao et al.(2008) that ginsenosides, Rh2, CK, and all sapogenins exhibited moderate CYP3A4 inhibition in baculosomes. According to Kong *et al*[21], the potency of a compound can be classified according to its *IC*_*50*_ values, as potent, if *IC*_*50*_≤ 20 μg/mL or ≤10 μM; moderate, if *IC*_*50*_ 20–100 μg/mL or 10–50 μM; or weak, if *IC*_*50*_≥100 μg/mL or ≥50 μM. CK is classified as a potent inhibitor of CYP3A4, a moderate inhibitor of CYP2C9, and a weak inhibitor of the other five CYPs tested in this study.

CYP2C9 is one of the most abundant CYP enzymes (about 20% of the hepatic total CYP content) [22]. It metabolizes a number of important clinical drugs, including anti-cancer, antibiotic, anti-diabetic, anti-epileptic, anti-hypertensive, anti-coagulant, and anti-hyperlipidemic drugs [23]. Of special interest is the CYP2C9-mediated metabolism of drugs with a narrow therapeutic index, such as warfarin and phenytoin [24]. Therefore, competing with the CYP2C9 metabolism can lead to severe toxicity. CYP3A4 is a major cytochrome P450 in human liver that has broad substrates [25]. CYP3A4 metabolizes not only xenobiotics, including the majority of drugs and carcinogens, but also many endogenous compounds, such as cholesterol, bile acids, fatty acids, prostaglandins, leukotrienes, retinoids, and biogenic amines, and thus most pharmacokinetic CAM–drug interactions involve CYP3A4[26]. The present study shows that CK is an inhibitor of both CYP2C9 and CYP3A4. Therefore, patients using CK in combination with conventional therapeutic drugs that are substrates of CYP2C9 and CYP3A4 for different reasons should be careful.

**Fig 10:**
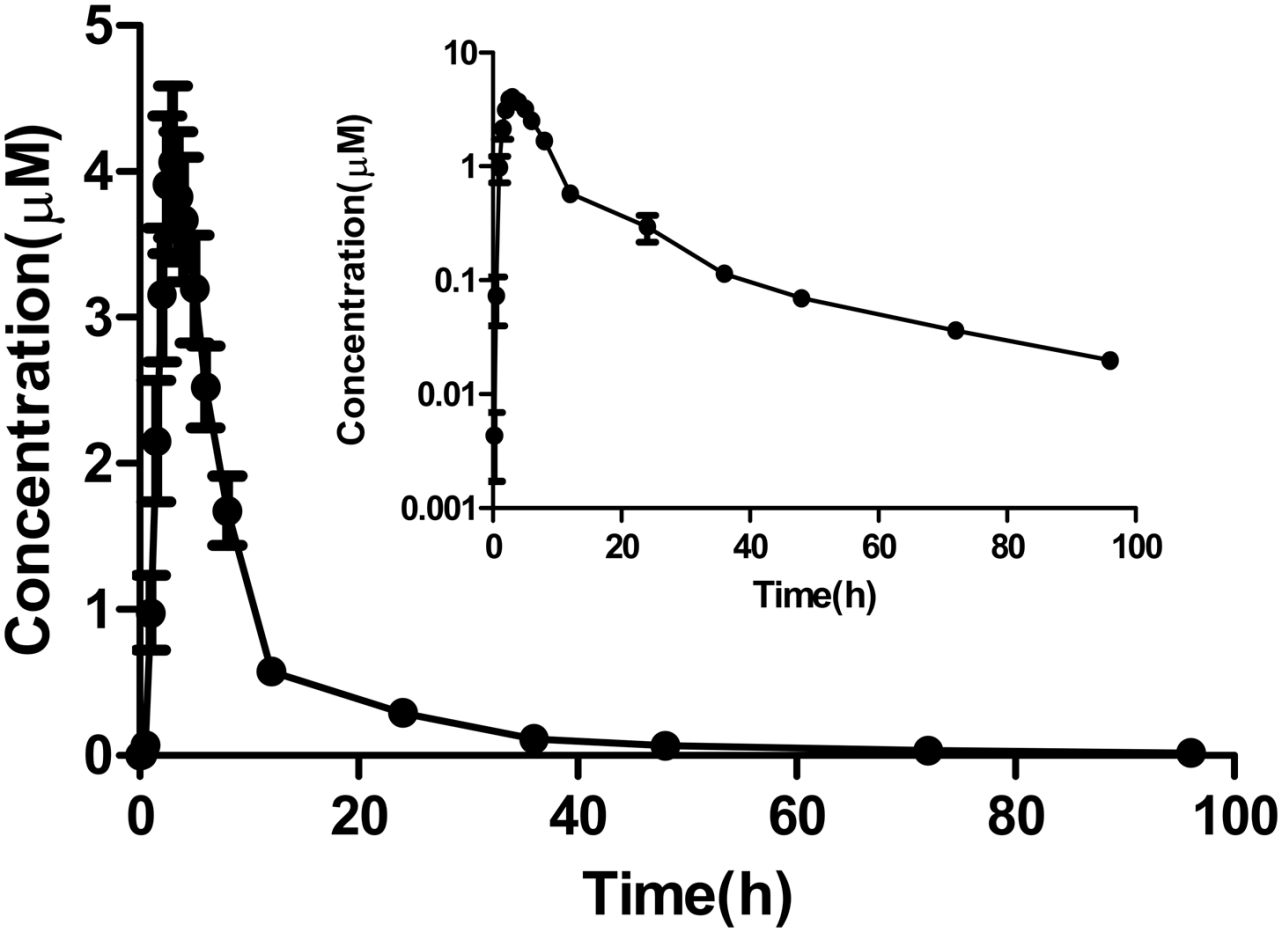
Plasma concentration –time profiles of CK in 10 healthy subjects after a single oral dose of 800 mg CK tablet. Inset depicts the same data on a semilogarithmic scale, each value is the mean value ± SD.

However, in contrast to the specific inhibitors of CYP2C9, the *IC*_*50*_ *and K*_*i*_ values of CK were 11-53-fold and 49-fold higher than those of sulfaphenazole in HLMs, respectively [15, 16]. For CYP3A4, the *IC*_*50*_ *and K*_*i*_ values of CK were 41-123-fold and 761-fold higher than those of ketoconazole in HLMs, respectively [17]. The potent inhibition of CK was poorer than that of enzyme-specific inhibitors. Presumably, the inhibiting potency of CK is too low to cause significant clinical CYP2C9 and CYP3A4 inhibition. Note that in our other study carried out in healthy volunteers, 10 healthy male volunteers received administrations of 800 mg pure CK tablets (100mg/tablet, provided by Zhejiang Hisun Pharmaceutical Company Limited, China) swallowed by 150 mL water, then series venous blood samples of 5 mL were collected into EDTA-containing tubes at 0, 0.25, 0.5, 1, 1.5, 2, 2.5, 3, 3.5, 4, 5, 6, 8, 12, 24, 36, 48, 72 and 96h. The plasma samples were analyzed by HPLC-MS/MS, the peak plasma concentration (C_max_) and time to peak plasma concentration (T_max_) were directly obtained from the observed concentration-time data. The area under the plasma concentration-time curve (AUC) from time zero to last measured concentration (AUC_(0−t)_) was calculated according to the linear trapezoidal rule, the estimate of oral clearance(CL/F) was calculated as CL/F = Dose /AUC_(0–t)_ [27]. The results showed the C_max_ of CK were 4.07 μM (95% CI: 2.89, 5.24). The C_max_ of CK of some volunteers were found to be near 5 μM through one-day supplementation of 800 mg, which is close to the *IC*_*50*_ and *Ki* values of CK for CYP2C9 and CYP3A4 (Fig 10). Thus, we still caution against CK–drug interaction despite its mildness.
